# Considering admixture when producing draft genomes: an example in North American ratsnakes (*Pantherophis alleghaniensis*/*Pantherophis obsoletus*)

**DOI:** 10.1093/g3journal/jkad113

**Published:** 2023-05-25

**Authors:** Frank T Burbrink, Sean M Harrington, Dean Bobo, Edward A Myers

**Affiliations:** Department of Herpetology, American Museum of Natural History, New York, NY 10024, USA; Department of Herpetology, American Museum of Natural History, New York, NY 10024, USA; INBRE Data Science Core, University of Wyoming, Laramie, WY 82071, USA; Institute for Comparative Genomics, American Museum of Natural History, New York, NY 10024, USA; Department of Herpetology, American Museum of Natural History, New York, NY 10024, USA; Department of Biological Sciences, Clemson University, Clemson, SC 29634, USA

**Keywords:** ratsnake, de novo genome assembly, *Pantherophis alleghaniensis*, *Pantherophis obsoletus*, admixture, hybridization

## Abstract

The number of reference genomes of snakes lags behind several other vertebrate groups (e.g. birds and mammals). However, in the last two years, a concerted effort by researchers from around the world has produced new genomes of snakes representing members from several new families. Here, we present a high-quality, annotated genome of the central ratsnake (*Pantherophis alleghaniensis*), a member of the most diverse snake lineage, Colubroidea. *Pantherophis alleghaniensis* is found in the central part of the Nearctic, east of the Mississippi River. This genome was sequenced using 10X Chromium synthetic long reads and polished using Illumina short reads. The final genome assembly had an N50 of 21.82 Mb and an L50 of 22 scaffolds with a maximum scaffold length of 82.078 Mb. The genome is composed of 49.24% repeat elements dominated by long interspersed elements. We annotated this genome using transcriptome assemblies from 14 tissue types and recovered 28,368 predicted proteins. Finally, we estimated admixture proportions between two species of ratsnakes and discovered that this specimen is an admixed individual containing genomes from the western (*Pantherophis obsoletus*) and central ratsnakes (*P. alleghaniensis*). We discuss the importance of considering interspecific admixture in downstream approaches for inferring demography and phylogeny.

## Introduction

The number of sequenced genomes of vertebrates has increased over the last decade. Hundreds of assembled genomes for birds and mammals are now available (Feng *et al*. 2020; Zoonomia Consortium 2020). However, for other vertebrate lineages, fewer genomic resources exist. Snakes are a diverse group of vertebrates with ∼4,000 named species, yet genomic resources for this group lag behind most other vertebrate clades. For example, only 42 species have de novo sequenced and assembled genomes (∼1% of species) across 10 families (∼27% of families). Dangerously venomous snakes represent the majority of sequenced genomes (37% Viperidae; 29% Elapidae) largely due to their importance in human medicine (Kishida *et al*. 2019; Suryamohan *et al*. 2020; Almeida *et al*. 2021; Li *et al*. 2021; Margres *et al*. 2021; Myers *et al*. 2022; Zhang *et al*. 2022).

In contrast, only six species within the family Colubridae (sensu Burbrink *et al*. 2020), representing 785 taxa, have publicly available genomes. Four of these genomes are from the New World tribe Lampropeltini (Pyron and Burbrink 2009; Chen *et al*. 2017) representing the glossysnake (*Arizona elegans*; Wood *et al*. 2022), the gophersnake (*Pituophis catenifer*), the cornsnake (*Pantherophis guttatus*; Ullate-Agote *et al*. 2014), and the western ratsnake (*Pantherophis obsoletus*; Ullate-Agote and Tzika 2021).

One of the most well-studied groups in this tribe are the North American ratsnakes (*P. obsoletus* complex). This species complex has been used to understand behavior, ecology, conservation, phylogenetics, and speciation (Fitch 1963; Burbrink 2001; Blouin-Demers and Weatherhead 2002; Blouin-Demers *et al*. 2005; DeGregorio *et al*. 2016; Burbrink *et al*. 2021). It represents four closely related species (*Pantherophis bairdi*, *Pantherophis obsoletus*, *Pantherophis alleghaniensis*, and *Pantherophis quadrivittatus*) originating in the late Miocene/early Pliocene, with an initial speciation event occurring at the Mississippi River (Burbrink *et al*. 2021). Given the importance of this species for examining processes on speciation and historical demography, it will be useful to have an annotated genome.

**Fig. 1.**
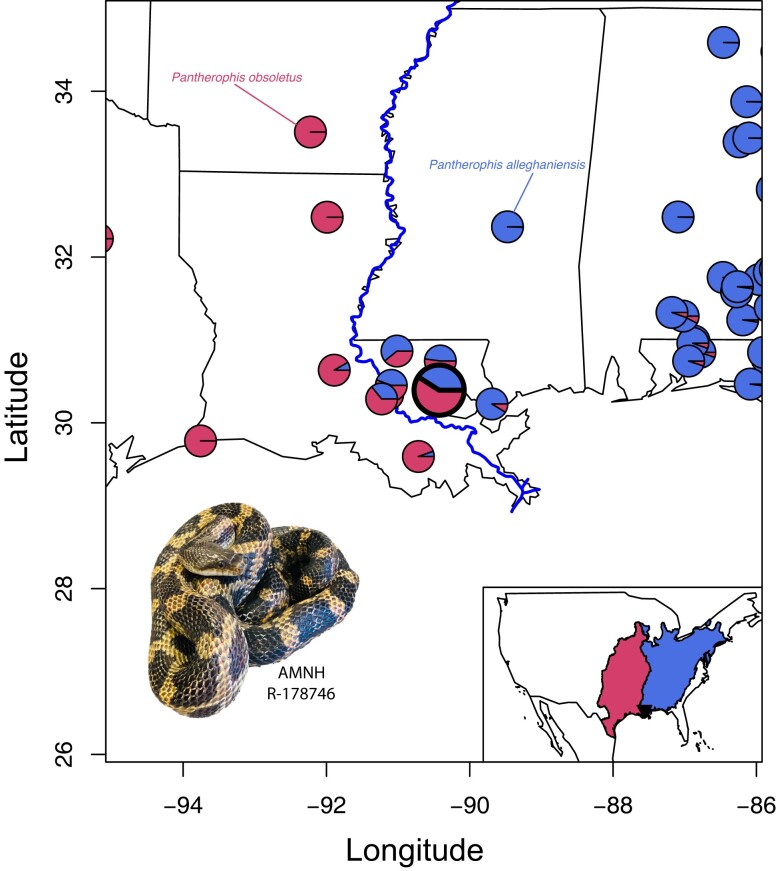
Map showing the admixture proportions between *Pantherophis alleghaniensis* and *Pantherophis obsoletus* in the lower Mississippi River. Note that the sample used for PanAll1.0 (AMNH R-17876) is enlarged with a bold border. Inset map showing the ranges of *P. alleghaniensis*/*P. obsoletus.* Photograph of *P. alleghaniensis*/*P. obsoletus* (AMNH R-17876) by FTB.

Here, we present a high-quality, annotated genome of a wild-caught specimen from the *P. obsoletus* complex (Fig. 1). We estimate admixture using ultraconserved elements (UCEs) from our previous study (Burbrink *et al*. 2021) between two species in this complex that meet in this region where this sample was taken. We discuss the basic features of the genome in this species and stress the importance of considering admixture when selecting specimens to be sequenced as representative genomic resources. This assembly will serve as a reference genome for the North American ratsnakes and permit future genomic and phylogenetic comparisons among ratsnakes, colubroids, snakes, and squamates in general.

## Materials and methods

### Specimen collection

This sample was collected in Tangipahoa Parish, Louisiana (30.394089, −90.427580) under a log, bordering cypress-tupelo swamp with mixed marsh habitat including red maple, wax myrtle, and red bay on 2018 March 15 by the lead author with permit holder Dr. Brian Crother, Oliver Ljustina, and Zach Nikolakis. The animal was euthanized following humane animal care protocals protocols conforming to recommendations from the Society for the Study of Reptiles and Amphibians (https://ssarherps.org/wp-content/uploads/2014/07/guidelinesherpsresearch2004.pdf). Blood was drawn from the caudal vein for genome sequencing, and the following tissues were sampled and flash frozen for RNA sequencing: brain, tongue, eye, heart, lung, skeletal muscle, skin, stomach, small intestine, liver, pancreas, spleen, kidney, and testes. The specimen and tissues have been accessioned at the American Museum of Natural History (AMNH R-178746). We extracted DNA from blood using the MagAttract HMW DNA Kit (QIAGEN; Hilden, Germany) and extracted RNA following the RNeasy Kit (QIAGEN; Hilden, Germany).

### Sequencing and assembly

The transcriptome for each organ was prepped using Illumina TruSeq RNAseq library kit for standard (non-stranded) sequencing at GENEWIZ (South Plainfield, NJ, USA) and sequenced on an Illumina NovaSeq 6000. Genome sequencing was performed at the NYGenome Center (New York, NY, USA) after Chromium 10X (10X Genomics) and TruSeq (Illumina) library preps. The assembled genome from AMNH R-178746 is referred to from here on as PanAll1.0.

High molecular weight genomic DNA fragments >30 kb were used as input into the Chromium 10X workflow. Sample indexing and partition barcoded libraries were prepared using the Chromium Genome Library and Gel Bead Kit (10X Genomics) according to manufacturer's protocols. The Chromium Controller was used to combine a library of 10X Genome Gel Beads with high molecular weight template genomic DNA (0.625 ng), a master mix of enzymes and buffer, and partitioning oil to create droplets containing single gel beads and DNA. During the process, genomic DNA was partitioned across approximately 1 million 10X gel beads-in-emulsion (GEM). The emulsion containing the GEM partitioned reactions was isothermally incubated (for 3 h at 30°C; for 10 min at 65°C; held at 4°C), and barcoded fragments ranging from a few to several hundred base pairs were generated. After amplification, the entire emulsion was collected, and GEMs were broken. Finally, the recovered barcoded DNA was size selected (450 bp) for library preparation. Illumina-specific sample indexing was added to the barcoded fragments to generate libraries according to the manufacturer's instructions. The barcode sequencing libraries were then quantified by qPCR (KAPA Biosystems Library Quantification Kit for Illumina platforms). Sequencing was conducted on the Illumina HiSeq X platform with 2 × 150 bp, paired-end reads based on the manufacturer's protocols. The resulting reads were then used as input to Supernova v2.0.1 (Weisenfeld *et al*. 2017), and a pseudo-haploid representation of the assembly was generated using the subcommand mkoutput.

To correct errors and fill gaps in the 10X assembly, we also generated 1 whole-genome shotgun sequencing library from AMNH R-178746 using the TruSeq DNA PCR-free library preparation kit (Illumina) following the manufacturer's instructions. Briefly, 1 μg of DNA was sheared using a Covaris LE220 sonicator (Adaptive Focused Acoustics). DNA fragments underwent bead-based size selection (450 bp) and were subsequently end-repaired, adenylated, and ligated to Illumina sequencing adapters. Final libraries were quantified using the Qubit Fluorometer (Life Technologies) or Spectromax M2 (Molecular Devices) and Fragment Analyzer (Advanced Analytical) or Agilent 2100 BioAnalyzer. Libraries were sequenced on an Illumina HiSeq X sequencer using 2 × 150-bp cycles.

Reads from the shotgun library was screened for adapter sequences and low-quality bases (Q < 10), which were trimmed using Cutadapt 1.8.1 (Martin 2011). Following this process, read-pairs that had any of the two ends shorter than 50 bp were discarded, and the remaining reads were mapped against phiX, using GEM mapper (edit distance ≤10%; Marco-Sola *et al*. 2012) for spike-in filtering. PCR-free reads were finally error-corrected using Lighter v1.1.1 (k = 21; Song *et al*. 2014). Processed PCR-free data were then used to produce the ABySS 2.0.2 (Jackman *et al*. 2017) shotgun assembly, exploring different k-mer sizes (37, 47, 57, 67, 77, 87, 97, 107, and 117). Flanks of decreasing size (starting at 1 kb down to 100 bp, in decrements of 100 bp) around each gap of the 10X assembly were searched in this shotgun assembly using GEM mapper. When both flanks mapped unambiguously, in the same contig and in the correct order and orientation, the sequence between the outermost mapping coordinates was extracted and used to patch the gap, giving priority to sequences originating from shotgun assembly of larger k-mer size. Remaining gaps in the 10X assembly were filled using the shotgun assembly with Compass (https://github.com/nygenome/compass), exploring multiple k-mer sizes (37, 47, 57, 67, 77, 87, 97, 107, and 117). All 10X scaffolds shorter than 200 kb were searched against the 10X assembly, using MegaBLAST (Zhang *et al*. 2000). Because Supernova v2.0.1 may produce duplicated contigs/scaffolds, scaffolds that fully aligned to a larger scaffold (coverage = 100%, identity ≥99%) were considered redundant and removed. Gene completeness of the final assembly was calculated using CEGMA 2.5 (Parra *et al*. 2007) with the default 248 core eukaryotic gene set and BUSCO 5.2.2 (Simão *et al*. 2015) with the Sauropsida gene set. We further scaffolded this assembly into pseudo-chromosomes using the *Thamnophis elegans* (NCBI assembly GCF_009769535.1) genome as a reference with RagTag v1.1.1 (Alonge *et al*. 2022) with default settings.

### Genome annotation

Prior to annotation, we soft masked the genome. We used RepeatModeler v4.1.0 (Flynn *et al*. 2020) to build repeat libraries. We then used RepeatMasker v4.1.0 (Smit *et al*. 2015) to iteratively mask the genome using the repeat library we generated as well as four other repeat libraries, following an established protocol for snake genome annotation (Card *et al*. 2019). These 4 libraries are as follows: (1) Tetrapoda from Repbase (Jurka *et al*. 2005; https://www.girinst.org/repbase/), and from Card *et al*. (2019), (2) a curated BovB/CR1 line library (file S15 in Card *et al*. 2019), (3) a library of known snake repeats (file S16 in Card *et al*. 2019), and (4) a library of unknown snake repeats (file S17 in Card *et al*. 2019) available in Figshare (https://figshare.com/articles/dataset/Data_including_genome_annotation_files_accompanying_Card_et_al_Genomic_basis_of_convergent_island_phenotypes_in_boa_constrictors/9793013).

We generated RNA-Seq data from 15 tissues and applied the following bioinformatic pipeline to each sample. We used Rcorrector 1.0.4. (Song and Florea 2015), a k-mer-based error correction method, which identifies trusted k-mers using De Bruijn graphs, to find and correct random sequencing errors. TranscriptomeAssemblyTools (https://github.com/harvardinformatics/TranscriptomeAssemblyTools) was used to remove errors that cannot be fixed using Rcorrector. Trim Galore 0.6.7 (https://github.com/FelixKrueger/TrimGalore) was used to remove low quality ends, adapters, and short sequences. Trinity 2.12.0 (Grabherr *et al*. 2011) was then used to assemble transcript sequences for each tissue and then combined for all tissues. Gene completeness was calculated on these assembled transcriptomes using BUSCO 5.2.2 (Simão *et al*. 2015) with the sauropsida gene set.

To annotate the genome assembly, we used Braker 2.1.16 (Lomsadze *et al*. 2014; Hoff *et al*. 2016) to produce full gene structure annotations with the combined mRNAs using the flags –softmasking –verbosity=3 –cores 16 –useexisting. We used BLAST 2.12.0+ (Camacho *et al*. 2009) to match the predicted proteins from Braker with sequences in the non-redundant RefSeq protein database (NR database downloaded 2022 July 7) with the following flags: -num_threads 1, -max_target_seqs 50, -outfmt 13, -evalue 1e-5. These results were imported into OmicsBox 2.2 (Götz *et al*. 2008) to provide gene ontology (GO) annotations using Blast2Go 2.2 within this program. We also predicted protein functions using InterProScan 5.59-91.0 (Jones *et al*. 2014) using the following parameters:–cpu 8, –disable-precalc, –disable-residue-annot, –formats TSV, XML,GFF3, –goterms,–iprlookup,–pathways, –seqtype p. Blast2GO and InterProScan annotations were combined in OmicsBox and exported as a GFF file. We used AGAT v1.0.0 (Dainat 2022) to estimate basic statistics including number of genes, isoforms, and average gene, intron, and coding sequence (CDS) lengths.

### Admixture estimation

The sample used to generate the PanAll1.0 genome was collected east of the Mississippi River in Louisiana, which was recently discovered to be a hybrid zone between two deeply divergent species, *P. obsoletus* and *P. alleghaniensis* (Burbrink *et al*. 2021). Using HZAR (Derryberry *et al*. 2014) to estimate the width of the hybrid zone for each UCE locus, Burbrink *et al*. (2021) identified diagnostic loci between these two species with hybrid zone widths below 130 km. We therefore pulled 2,500 bp of sequence around UCE baits from the PanAll1.0 assembly using the Phyluce commands phyluce_probe_run_multiple_lastzs_sqlite and phyluce_probe_slice_sequence_from_genomes (Faircloth 2016). Taking the top 20 diagnostic UCEs from 136 individuals representing *P. obsoletus* and *P. alleghaniensis* from Burbrink *et al*. (2021), we aligned those same PanAll1.0 UCEs using muscle (Edgar 2004) and trimmed these to the length of the UCE loci from Burbrink *et al*. (2021). We used TESS3r 1.1.0 (Caye *et al*. 2016) and sparse nonnegative matrix factorization (SNMF; Frichot *et al*. 2014) in R (R Core Team 2010) with the function *tess* applying the least squares method, *projected.ls*, using 100 iterations to estimate groupings and ancestral admixture coefficients for AMNH R-178746.

## Results and discussion

### Genome assembly

**Fig. 2.**
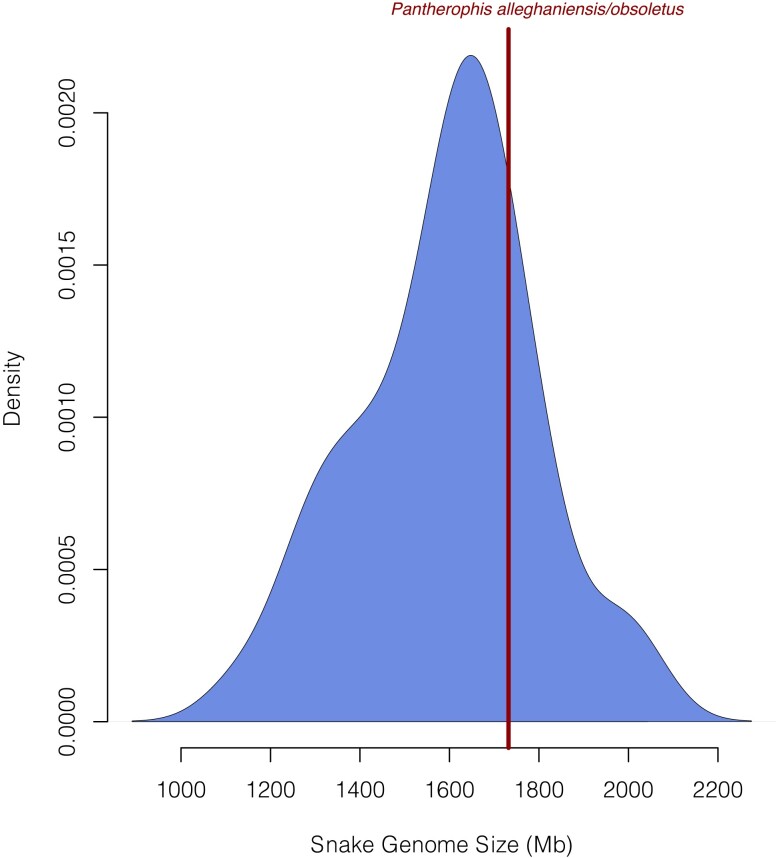
Distribution of snake genome sizes from 43 whole genomes and *Pantherophis alleghaniensis*/*Pantherophis obsoletus* (AMNH R-17876) shown in dark red (1.73 Gb). Data for genomes collected from Castoe *et al*. (2011), Vonk *et al*. (2013), Gilbert *et al*. (2014), Ullate-Agote *et al*. (2014), Aird *et al*. (2017), Pasquesi *et al*. (2018), Shibata *et al*. (2018), Kishida *et al*. (2019), Burbrink *et al*. (2020), Suryamohan *et al*. (2020), Almeida *et al*. (2021), Gower *et al*. (2021), Köhler *et al*. (2021), Margres *et al*. (2021), Rhie *et al*. (2021), Ullate-Agote and Tzika (2021), Grismer *et al*. (2022), Myers *et al*. (2022), Wood *et al*. (2022), and Zhang *et al*. (2022) and see https://www.ncbi.nlm.nih.gov/assembly/? term=txid8570[Organism:exp].

The assembly of PanAll1.0 generated 65,651 scaffolds from 92,413 contigs. After gap patching with the shotgun reads and gap filling using Compass, we filled 45,559 and 26,424 gaps, respectively. After the removal of redundant contigs/scaffolds, we were left with 26,850 gaps. The final, scaffolded genome had a L50 of 22 scaffolds with an N50 of 21.818 Mb, whereas the pre-scaffolded genome had an L50 of 2,745 contigs and an N50 of 153.585 kb. The maximum scaffold length was 82.078 Mb, and maximum contig length was 1.686 Mb. We estimated that Chromium 10X and shotgun TruSeq coverage were 69.5× and 68.3×, respectively. The estimated genome size from Supernova of this genome was 1.73 Gb, which is larger than the average of the currently sequenced 42 snake genomes (X = 1.59 Mb; SD = 2.0; range 1.13–2.20 Gb; see Fig. 2). Within the genus *Pantherophis*, our estimate is only 19 Mb larger than the cornsnake (*P. guttatus*) genome and 33 Mb greater than the previously sequenced ratsnake genome (*P. obsoletus*; Ullate-Agote *et al*. 2014; Ullate-Agote and Tzika 2021). We note that the previous two species of *Pantherophis* sequenced were not representative of wild-caught animals. The PanAll1.0 assembly had a GC content of 40.5%, which is close to the values for *P. guttatus* and *P. obsoletus* (40.8 and 39.1, respectively) and similar to the average for all snakes (mean = 38.6; SD = 6.03). This 10X assembly was successfully scaffolded to the *T. elegans* genome. A total of 135 Mb of sequence were not scaffolded to this chromosome length assembly, this however was expected given the estimated genome sizes of these two taxa where our *P. alleghaniensis* genome size was 1.73 Gb, and the *T. elegans* size was estimated to be 1.67 Gb. We also generated paired reads for 14 tissues, though failing to produce contigs for brain tissue (see Supplementary Table 1 for information on mean and median contig length, contig N50, and number of paired reads).

### Genome annotation

**Fig. 3.**
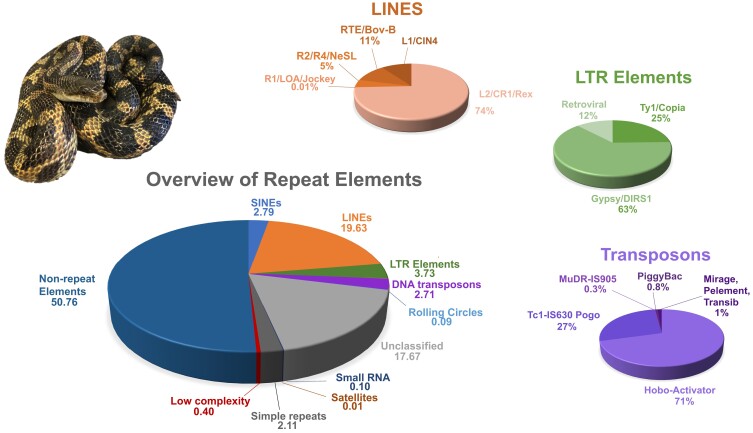
Overview proportion of the genome representing repeat elements and types of repeat elements, with details for LINEs, LTR elements, and transposons included. Photograph of *Pantherophis alleghaniensis*/*Pantherophis obsoletus* (AMNH R-17876) by FTB.

We estimated that 49.24% of the PanAll1.0 genome represent repeat elements (Fig. 3). This is within the known range of repeats for squamates (25–73%; Pasquesi *et al*. 2018). Previous estimates of repeat elements found similar values in *P. obsoletus* (45.18%) and *P. guttatus* (39.1%; Ullate-Agote *et al*. 2014; Ullate-Agote and Tzika 2021). We found a large diversity of repeat elements in PanAll1.0 characteristic of squamates (Pasquesi *et al*. 2018). These repeats are dominated by long interspersed elements (LINEs; 19.63%) followed by long terminal repeat elements (LTR; 3.73%) and short interspersed nuclear elements (SINEs; 2.79%). LINEs are mostly composed of L2/CR1/Rex elements (74%), LTR elements by Gyspsy/DIRS1 (63%), and transposons by Hobo-Activator (71%).

**Fig. 4.**
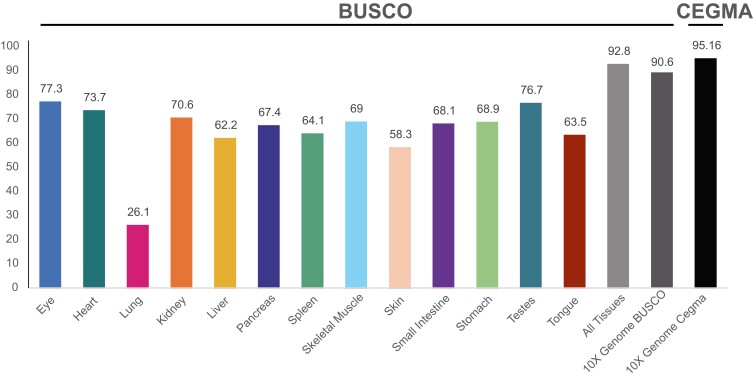
Percentage of sauropsida BUSCO loci found for each tissue type, combined tissues, and the 10X Genome. The Cegma loci percentages for the 10X genome are also included.

Using the Sauropsida BUSCO data set, we found that the genome assembly is relatively complete (90.6%) and for CEGMA was 95.16%. From our transcriptome data sets, complete BUSCO scores ranged from 26.1% in the lung, up to 77.3% in the eye. Combining all tissue mRNAs, we found a high BUSCO score of 92.8% (Fig. 4).

We annotated the PanAll1.0 genome assembly using the combined mRNA data set. From this, AGAT calculated that Braker predicted 42,480 genes and 47,657 mRNAs (5,177 isoforms) of which 28,448 have functional annotations. We note that this was an excessively high number of predicted proteins. Many annotations from BRAKER were therefore likely spurious. We used the results of the functional annotation steps to perform quality control on the BRAKER results. We only considered predicted proteins that had BLAST or InterPro hits but also used an Annotated or Mapped status from Blast2Go/OmicsBox. We generated a BED file with the coordinates of those transcripts, then used BEDTOOLS to filter the BRAKER GTF file so only the annotations that passed this QC are included. Our final gene and annotation data set include 28,368 predicted transcripts/proteins, which is similar to previous estimates within this genus (24,107 for *P. obsoletus* and 24,258 for *P. guttatus*; Ullate-Agote *et al*. 2014; Ullate-Agote and Tzika 2021).

We estimated the following statistics across the genome (without isoforms in parentheses): The average gene length was 22,164 bp (22,164 bp), average mRNA length was 26,430 bp (22,099 bp), mean CDS length was 1,550 bp (1,396 bp), average exon length was 168 bp (174 bp), mean exons per CDS was 9.4 (8.2), mean introns per CDS was 8.2 (7.2), and mean intron length was 2,975 bp (2,903 bp).

### Admixture

Using the top 20 diagnostic UCE loci, we identified that AMNH R-178746 is indeed an admixed individual with estimated 59% *P. obsoletus* and 41% *P. alleghaniensis* ancestral coefficients. The sampling location of this specimen is in the general area of the hybrid zone where other individuals with similar admixture proportions are located (Fig. 1). We note that none of the UCEs in AMNH R-178746 contain heterozygous sites, and similarly 38% of samples from Louisiana also presented no heterozygous sites for the diagnostic loci. This suggests that while this population may be formed from the hybridization of two species, many generations of drift may have eliminated heterozygosity within these loci (Fitzpatrick 2012). Therefore, it is not likely that AMNH R-178746 is a recent hybrid (F1 or F2).

Although it is uncommon to report admixture proportions in de novo assembled genome papers, we believe that it is relevant particularly when estimating population-level processes or phylogenetic relationships. For example, methods that can use a single genome to generate estimates of effective population size through time such as Pairwise Sequential Markovian Coalescent (PSMC; Li and Durbin 2011) are important for understanding the demographic history of organisms across time periods that span major changes in the environment (Dyson *et al*. 2022). However, these methods assume that individual genomes are sampled from a panmictic population. These assumptions are potentially violated given varying degrees of admixture, particularly among organisms that diverged thousands of generations ago. Discoveries of admixture and introgression are becoming common with genome-scale data and have revealed that gene flow is quite common between animal species (Rundle and Nosil 2005; Harrison and Larson 2014; Mallet *et al*. 2016; Ottenburghs 2020; Burbrink and Ruane 2021; Myers 2021). In cases where population genetic or phylogeographic data exist or can be generated for the taxon of interest to produce locations of hybrid zones, researchers are then encouraged to sample individuals away from zones of contact where admixture is expected to be high. We also suggest that basic inferences of admixture be reported with genome assemblies when possible, particularly where samples are taken on the edge of ranges between closely related species or when deep phylogeographic structure is known to exist.

### General findings

Here, we present an annotated draft genome of the western and central ratsnake (*P. obsoletus*/*P. alleghaniensis*) predicting 28,368 genes (including isoforms) using mRNA sequences from 14 tissue types. Of these, 24,274 (85.6%) have known functional descriptions, and PanAll1.0 therefore provides a useful reference genome for colubroid snakes.

We also demonstrate the amount of admixture between closely related species present in a draft genome sequence. Here, the deepest divergence within the North American ratsnake complex occurs in the Mississippi River. This divergence between *P. obsoletus* and *P. alleghaniensis* occurred ∼3.1 MYA. Just east of the Mississippi River, there is now a known hybrid zone between *P. obsoletus* and *P. alleghaniensis* (Burbrink *et al*. 2021). Our estimates using UCEs with a previous data set predict that the sequenced individual (assembly PanAll1.0) likely represents admixture between *P. obsoletus* and *P. alleghaniensis* occurring many generations ago. This study highlights the importance for examining admixture in reference genomes, given how widespread introgression is at hybrid zones and even among anciently diverged taxa (Harrison and Larson 2014; Barth *et al*. 2020; Burbrink and Ruane 2021; Myers 2021).

We underscore that using downstream analyses to investigate population demography in a coalescent framework using single genomes may produce biased results when assuming a sample originated from a panmictic population (Kingman 1982; Wakeley 2008; Mather *et al*. 2020). Therefore, as more genomes are sequenced for population or phylogeographic studies, researchers should leverage these samples to examine the degree and location of admixture in their target genomes to better understand speciation processes related to introgression. Future studies using this reference genome (PanAll1.0), combined with additional genomic and morphological data, will further help define the shapes of hybrid zones and understand which genes were key for initial divergence and maintenance of species boundaries between these taxa.

## Supplementary Material

jkad113_Supplementary_Data

## Data Availability

The following data are available as follows: (1) Raw Chromium 10X, TruSeq, and RNA-Seq data are hosted on the NCBI SRA: SAMN32907824 and PRJNA927268, (2) assembled and scaffolded Chromium 10X and TruSeq fasta are available on NCBI, JAQZSL000000000, and BioProject accession number PRJNA926953, and (3) GFT files, Chromium 10X and TruSeq data (unscaffolded and scaffolded), transcriptome data, and diagnostic UCEs are available on Figshare: 10.6084/m9.figshare.21947927. Supplemental material available at G3 online .
