# Supplementary material for: Considering admixture when producing draft genomes: an example in North American ratsnakes (Pantherophis alleghaniensis/Pantherophis obsoletus)
Source: G3 (Bethesda). 2023 May 25;13(8):jkad113. doi: 10.1093/g3journal/jkad113 (PMC10411579; doi:10.1093/g3journal/jkad113)
Supplement: jkad113_Supplementary_Data [file jkad113_supplementary_data.docx]

Supplemental Table 1. Statistics for transcriptomes for each organ using RNA-seq. including mean and median contig length, contig N50 and number of paired reads.

| **Organ** | Mean contig length | Median contig length | Contig N50 | Paired Read Count |
| --- | --- | --- | --- | --- |
| **kidney** | 884.52 | 420 | 1770 | 108974623 |
| skin | 803.11 | 424 | 1410 | 136260626 |
| pancreas | 740.55 | 349 | 1449 | 84473904 |
| tongue | 849.51 | 427 | 1590 | 106533475 |
| Small intestine | 937.5 | 452 | 1854 | 84834993 |
| heart | 850.7 | 392 | 1747 | 113346383 |
| eye | 974.44 | 441 | 2069 | 104895441 |
| spleen | 780.72 | 398 | 1394 | 107586283 |
| stomach | 884.77 | 430 | 1718 | 113542882 |
| liver | 914.29 | 434 | 1824 | 100925890 |
| testes | 932.93 | 444 | 1871 | 92861824 |
| skeletal-muscle | 1040.01 | 473 | 2155 | 93640090 |
| lung | 464.06 | 279 | 539 | 28012295 |
| brain | NA | NA | NA | 324032844 |
| All organs | 863.88 | 412 | 1702 | 1178825737 |
